# Resveratrol Metabolites Are Able to Reduce Steatosis in Cultured Hepatocytes

**DOI:** 10.3390/ph13100285

**Published:** 2020-09-30

**Authors:** Jenifer Trepiana, Stéphanie Krisa, Elodie Renouf, María Puy Portillo

**Affiliations:** 1 Nutrition and Obesity Group, Department of Nutrition and Food Science, Faculty of Pharmacy, University of the Basque Country (UPV/EHU), and Lucio Lascaray Research Centre, 01006 Vitoria-Gasteiz, Spain; jenifer.trepiana@ehu.eus; 2 BIOARABA Institute of Health, 01009 Vitoria-Gasteiz, Spain; 3 CIBEROBN Physiopathology of Obesity and Nutrition, Institute of Health Carlos III (ISCIII), 28029 Vitoria-Gasteiz, Spain; 4 Université de Bordeaux, UR Œnologie, MIB, EA 4577, USC 1366 INRA, 33882 Villenave d’Ornon, France; stephanie.krisa@u-bordeaux.fr (S.K.); elodie.renouf@u-bordeaux.fr (E.R.); 5 Polyphénols Biotech (Technology Transfer Unit), ISVV, 33882 Villenave d’Ornon, France

**Keywords:** resveratrol metabolites, *trans*-resveratrol-4′-*O*-glucuronide, *trans*-resveratrol-3-*O*-glucuronide, *trans*-resveratrol-3-*O*-sulfate, dihydroresveratrol, liver steatosis, AML12 hepatocytes

## Abstract

Steatosis is characterized primarily by excessive lipid accumulation in the form of triglycerides in the liver. Although resveratrol shows a low bioavailability, it has significant positive effects on steatosis. The aim of this study was to analyze whether some phase II and microbial resveratrol metabolites (*trans*-resveratrol-4′-*O*-glucuronide (R-4G); *trans*-resveratrol-3-*O*-glucuronide (R-3G); *trans*-resveratrol-3-*O*-sulfate (R-S) and dihydro-resveratrol (DH-R) were effective in reducing hepatocyte fat accumulation. An in vitro model mimicking the hepatocyte situation in fatty liver was developed by incubating mouse AML12 hepatocytes with palmitic acid (PA). For cell treatments, hepatocytes were incubated with 1, 10, or 25 µM resveratrol or its metabolites. Triglycerides and cell viability were assessed using commercial kits. Protein expression of enzymes and transporters involved in triglyceride metabolism were analyzed by western blot. We show for the first time that resveratrol and all the tested metabolites, at 1 µM, partially prevented lipid accumulation induced by the saturated fatty acid PA in AML12 hepatocytes. This effect was mainly due to the inhibition of *de novo* lipogenesis. This demonstrates that the low bioavailability of resveratrol is not as big a problem as it was thought to be, because resveratrol metabolites contribute to the delipidating effects of the parent compound.

## 1. Introduction

In recent decades, phenolic compounds have become an emerging field of interest in nutrition research. These molecules show an aromatic structure, with one or more hydroxyl groups, classified into several families according to the nature of their carbon skeleton. Among them, stilbenes are promising compounds due to their beneficial effects on health [1].

Resveratrol (*trans*-3,5,4-trihydroxystilbene) is the most extensively studied stilbene. It is produced in plants defense mechanism in response to stress. The scientific community has set its sights on a great number of studies on this molecule, due to its wide range of positive biological effects [2]. In spite of this, an important limitation to using resveratrol as a tool for the management of chronic diseases is its low bioavailability. This is due to the extensive phase II metabolism that happens in intestine and liver [3,4,5]. In view of this problem, different nanotechnology approaches (microparticles, liposomes, nanocapsules, etc.), have been developed over the last few years to increase resveratrol bioavailability [6].

Pharmacokinetic studies have revealed that the bioavailability of resveratrol found in plasma and tissues after oral administration is less than 1% [4]. This occurs due to the rapid metabolism of resveratrol into sulphate and glucuronide conjugates [7]. Consequently, the concentration of these conjugates is higher than that of the parent compound [8,9,10]. Several authors have reported that, after an oral administration in the range of mg, the plasma concentration of resveratrol was in the nanomolar range, whereas metabolite concentrations were in the micromolar range [9,11,12]. In addition to phase II metabolism, the remaining resveratrol that has not been absorbed in the intestine suffers microbial metabolism in the colon, consisting of the cleavage of hydroxyl groups or a hydrogenation. Dihydroresveratrol has been identified as the first microbial-derived metabolite [9,13,14], and *Slackia equolifaciens* and *Adlercreutzia equolifaciens* as the main bacteria involved in its production [15].

In this context, studies devoted to analyzing the bioactivity of resveratrol metabolites and thus their potential contribution to the beneficial health effects of the parent compound are needed. Although, so far, little is known concerning this issue, several studies have shown that resveratrol metabolites show positive effects in cancer [16,17,18], neurodegenerative diseases [19], and type 2 diabetes mellitus [20], as well as exhibiting anti-inflammatory actions [21]. In previous studies, we showed for the first time that several metabolites displayed delipidating effects in adipocytes, and that they modified adipokine production [22,23,24].

The present study focuses on non-alcoholic liver steatosis, which consists of an excessive accumulation of lipids as triglycerides and cholesterol in the liver, in the absence of excessive alcohol intake [25]. With regard to this metabolic alteration, it has been reported that resveratrol prevents its development and progression in rodent models [26,27,28,29,30,31,32], and controversial results have been reported in human studies to date [33,34,35,36,37,38,39,40]. However, as far as we know, there is no data available concerning the effects of resveratrol metabolites on this metabolic disorder.

Hence, the aim of the present study was to analyze whether the following phase II resveratrol metabolites, *trans*-resveratrol-4′-*O*-glucuronide (R-4G), *trans*-resveratrol-3-*O*-glucuronide (R-3G), and *trans*-resveratrol-3-*O*-sulfate (R-S), and the microbial resveratrol metabolite dihydroresveratrol (DH-R) showed any beneficial effect in the prevention of hepatic steatosis, using AML12 hepatocytes.

## 2. Results

### 2.1. Effects on Triglyceride Accumulation

**Figure 1:**
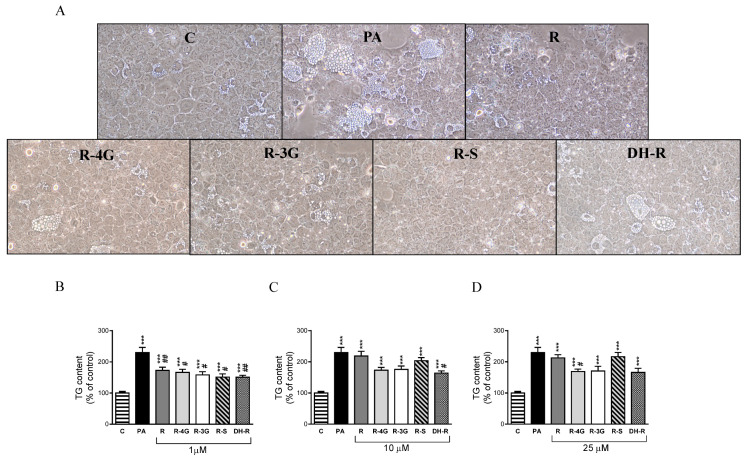
Optical microscopy images showing structural features at 1 µM (**A**) and triglyceride content in AML12 hepatocytes exposed to 0.3 M palmitic acid (PA) with or without resveratrol (R) or its metabolites (R-4G, R-3G, R-S and DH-R) at 1 µM (**B**), 10 µM (**C**) or 25 µM (**D**) for 18 h. Data are means ± SEM. *** *p* < 0.001 vs. control group. # *p* < 0.05 and ## *p* < 0.01 vs. PA group.

To examine the effects of resveratrol and its metabolites, optical analysis was carried out. In addition, in order to obtain more accurate results, the amounts of triglyceride accumulated in the hepatocytes were spectrophotometrically quantified. Optical microscopy analysis showed that treatment with PA alone displayed macrovacuolar steatosis in AML12 hepatocytes, whereas in general terms, coincubation with resveratrol metabolites (1 µM) presented smaller scattered fat vacuoles in the cytoplasm of the AML12 (Figure 1a). Moreover, triglyceride quantification showed that at 1 µM, resveratrol and all the metabolites partially prevented the accumulation of triglycerides induced by PA (R—25%; R-4G—28%; R-3G—31%; R-S—34%; DH-R—34% vs. PA group; Figure 1b). At higher concentrations, only 10 µM DH-R, and 25 µM R-4G significantly prevented the intracellular triglyceride accumulation (Figure 1c,d). These results indicate that the lowest concentration of resveratrol and its metabolites was the most effective in preventing triglyceride accumulation.

### 2.2. Cell Viability

**Figure 2:**
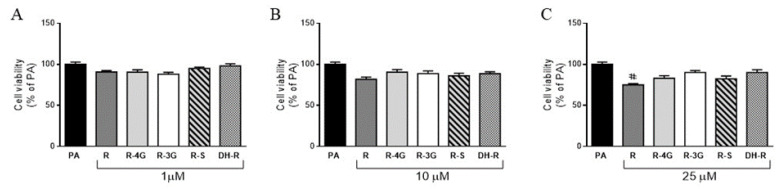
Cell viability in AML12 hepatocytes exposed to 0.3 M palmitic acid (PA) with or without resveratrol (R) or its metabolites (R-4G, R-3G, R-S and DH-R) at 1 µM (**A**), 10 µM (**B**) or 25 µM (**C**) for 18 h. Data are means ± SEM. # *p* < 0.05 vs. PA group.

Incubation of hepatocytes with resveratrol or resveratrol metabolites for 18 h did not decrease cell viability in the whole range of the concentrations studied, when compared to the PA group (Figure 2a–c). Only the supraphysiological concentration of the parent compound (25 µM) decreased hepatocyte viability when cells were co-incubated with PA (Figure 2c). Moreover, it is worth mentioning that incubation of AML12 hepatocytes with PA alone, at the dose used (0.3 mM), reduced the cell viability (−45%) when compared to the control group (non-steatotic cells; data not shown).

### 2.3. Resveratrol Metabolism into the Steatotic AML12 Cells and in the Incubation Cell Media

In order to evaluate the potential metabolism of resveratrol during the treatment, both the AML12 cells and the incubation cell media were analyzed at different times during the incubation period (1, 3, 6, 8, 16, 18 h). In this experiment, among the three resveratrol doses, only the highest (25 μM) was used, on the grounds that the sensitivity of our chromatographic technique did not allow us to detect resveratrol and its metabolites at all times when resveratrol is used at 10 and 1 µM. This was true mainly in cells.

**Figure 3:**
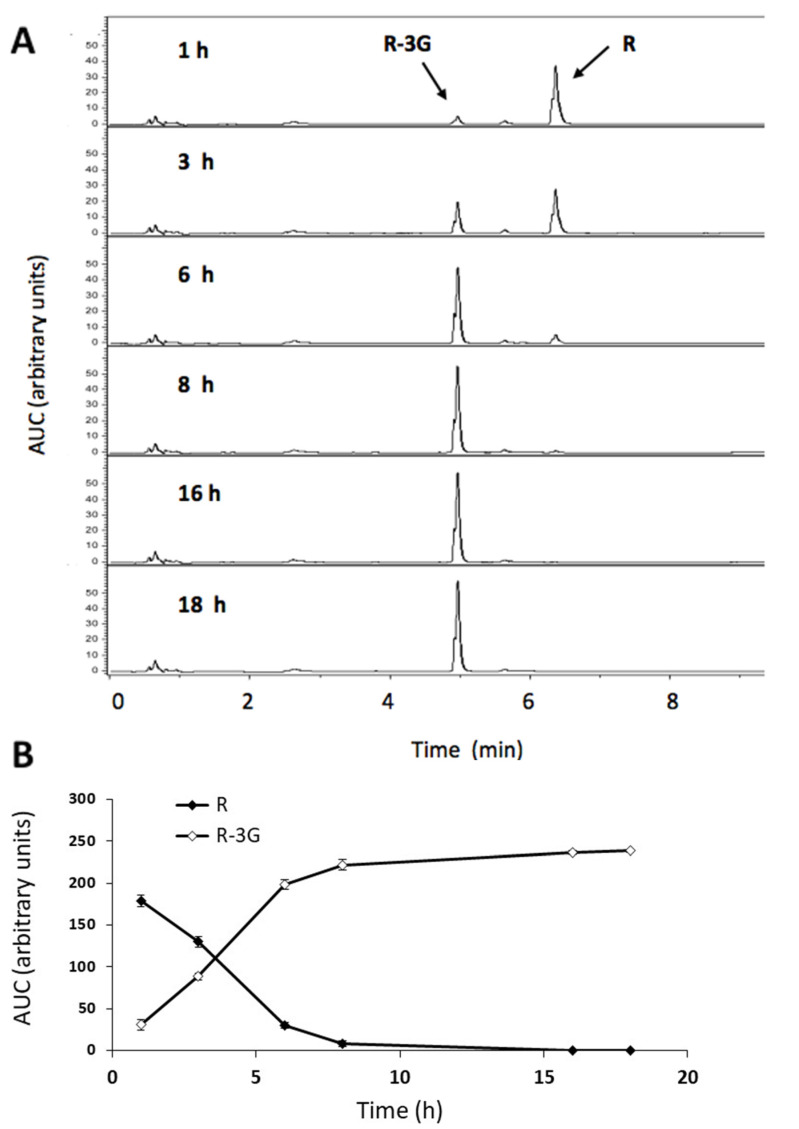
UHPLC-UV profile of resveratrol (R) and trans-resveratrol-3-*O*-β-D-glucuronide (R-3G) in the AML12 hepatocyte incubation media. The chromatograms show the metabolism of R into R-3G in the steatotic AML12 incubation media of cells exposed to 0.3 M palmitic acid (PA) and R (25 μM) at 1, 3, 6, 8, 16 and 18 h of incubation (**A**). Amount of R and R-3G in the AML12 hepatocytes incubation media over 18 h (**B**).

In the incubation medium, the amount of resveratrol decreased rapidly, as from 1 h and after 8 h of incubation, more than 95% of resveratrol was metabolized. UHPLC analysis highlights the presence of the R-3G metabolite in all samples (Figure 3a). In the incubation medium, the amounts of R-3G increased as a result of the lessening of resveratrol during the 6 h time lapse (Figure 3b). No other metabolite was detected in the samples.

**Figure 4:**
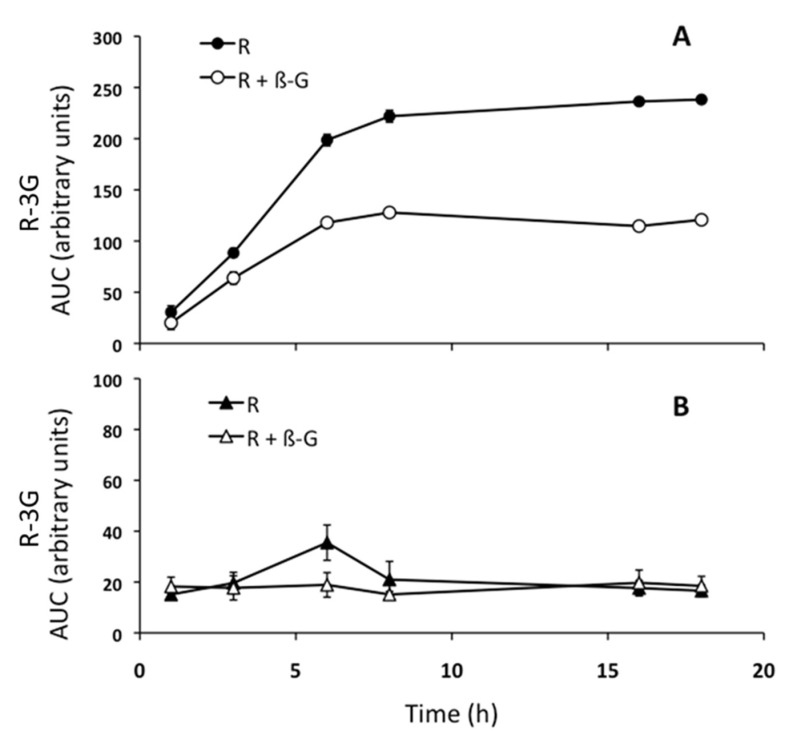
Amount of *trans*-resveratrol-3-*O*-β-D-glucuronide (R-3G) in the AML12 hepatocytes incubation media (**A**) and cells (**B**). As shown in the legend, AML12 cells were incubated with PA and resveratrol (25 µM), or with PA and resveratrol + β-glucuronidase (400 U/mL) for 1, 3, 6, 8, 16, and 18 h.

As the amount of resveratrol and its metabolites in the incubation medium depends on the metabolic activity of hepatocytes and the exchanges through the plasmatic membrane of hepatocytes, in order to better understand these aspects, steatotic AML12 cells were incubated with resveratrol (25 µM) in the presence, or not, of β-glucuronidase (400 U/mL). Thus, the area under the curve (AUC) of R-3G was plotted along the time. As shown in Figure 4a, the amount of R-3G reached a plateau at 8 h in the incubation medium, when hepatocytes were incubated with resveratrol alone. However, when steatotic AML12 hepatocytes were incubated with resveratrol and β-glucuronidase, the proportion of R3-G in medium at 18 h decreased 49% compared to the medium of cells incubated with resveratrol alone (Figure 4a). In addition, we had previously checked that after 18 h, resveratrol was still present in the incubation medium without cells, meaning that the phenolic compound was stable in this medium (data not shown).

In the cells incubated with resveratrol in the presence, or not, of β-glucuronidase, which catalyzes the hydrolysis of the glucuronides back to the parent compound, the R-3G was present at 1 h, and its quantity varied only a little until 18 h of incubation (Figure 4b). R-3G, therefore, quickly crossed the plasma membrane of hepatocytes to reach the culture medium after its production.

### 2.4. Resveratrol Delipidating Effects in the Presence or Absence of ß-glucuronidase

**Figure 5:**
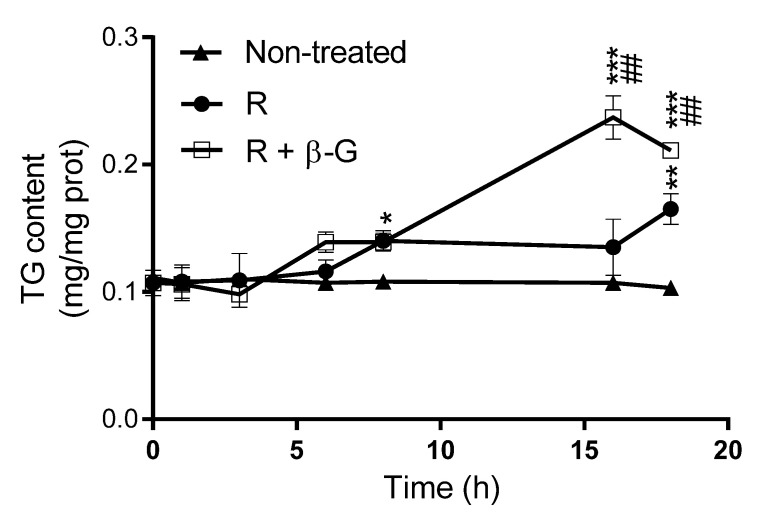
Triglyceride amounts in AML12 hepatocytes exposed, or not, to 0.3 M palmitic acid (PA) with resveratrol (R; 1 µM), in the presence or not of β-glucuronidase (β-G; 400 U/mL) over 0, 1, 3, 6, 8, 16, and 18 h. Data are means ± SEM. * *p* < 0.05, ** *p* < 0.01 and *** *p* < 0.001 vs. non-treated cells, and ## *p* < 0.01 vs. hepatocytes treated with resveratrol alone.

Triglyceride levels were measured in steatotic AML12 hepatocytes treated with resveratrol alone (1 µM), or with resveratrol + β-glucuronidase (400 U/mL) during the incubation period. As shown in Figure 5, and according to the results shown in Figure 4, the amount of triglycerides in hepatocytes incubated with PA and resveratrol, where the parent compound should be totally metabolized to R-3G after 8 h of incubation, was not significantly modified during the 18 h of incubation. This probably means that the increase in triglyceride accumulation induced by PA was compensated by the action of resveratrol and/or R-3G. However, when β-glucunoridase was added to the incubation medium, triglyceride accumulation was increased between 8 and 16 h (Figure 5). This means that, under this experimental condition, which is characterized by the presence of non-metabolized resveratrol, the effect of PA was not completely compensated by the phenolic compound.

### 2.5. Effects of Resveratrol and Resveratrol Metabolites on Proteins Involved in Triglyceride Metabolism

**Figure 6:**
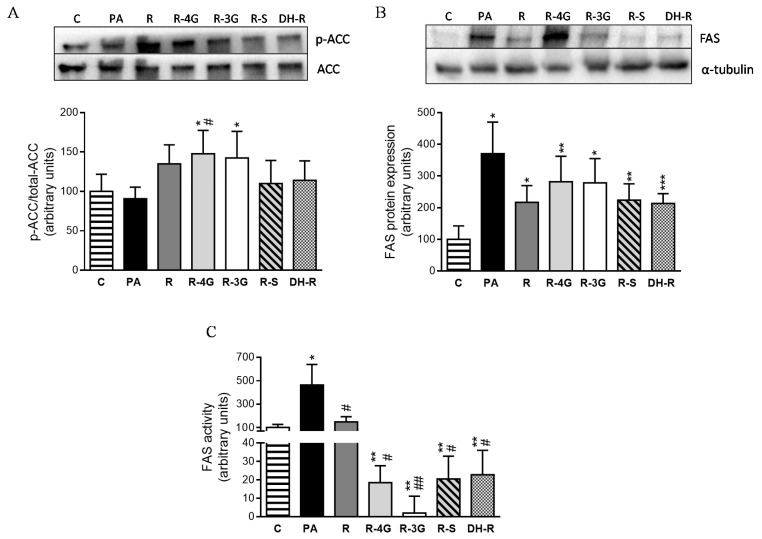
p-ACC/total-ACC ratio (index of ACC activity) (**A**), protein expression of FAS (**B**) and FAS activity (**C**) in AML12 hepatocytes incubated with 0.3 M palmitic acid (PA), with or without resveratrol or its metabolites (R-4G, R-3G, R-S and DH-R) at 1µM. The western blot bands shown are representative of 6 samples/group. Data are means ± SEM. * *p* < 0.05, and ** *p* <0.01 vs. the control cells. # *p* < 0.05 and ## *p* < 0.01 vs. PA group. ACC: Acetyl-CoA carboxylase; FAS; fatty acid synthase.

In order to elucidate the intracellular mechanisms involved in the delipidating effect of the analyzed molecules, the protein expression of key proteins involved in lipid metabolism were assessed by immunoblot. Taking into account that ACC is inactivated by phosphorylation, the ratio phosphorylated-ACC/total-ACC is considered as an index of ACC activity (lower ratio meaning the activation of the enzyme). Incubation of hepatocytes with the lowest concentration of resveratrol resulted in an increase in this ratio, which reached statistical significance in the case of R-4G and R-3G, meaning that ACC was inhibited (Figure 6a). With regard to fatty acid synthase (FAS), resveratrol and the metabolites tended to avoid the increase in their protein expression induced by PA, but statistical significance was not reached (*p* < 0.1; Figure 6b). In view of these results, FAS activity was also measured. Treatment with resveratrol (1 µM) for 18 h restored FAS activity, which reached the value observed in non-steatotic cells (Figure 6c). Furthermore, cells treated with resveratrol metabolites showed even lower FAS activity values than those of the untreated cells (Figure 6c).

**Figure 7:**
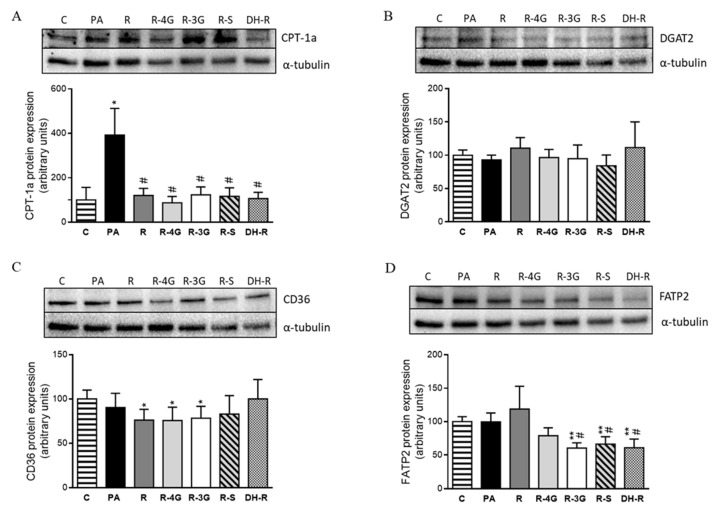
Protein expression of CPT-1a (**A**), DGAT2 (**B**), CD36 (**C**) and FATP2 (**D**) proteins in AML12 cells exposed to 0.3 M palmitic acid (PA) with or without resveratrol (R) or its metabolites (R-4G, R-3G, R-S and DH-R) at 1µM. The western blot bands shown are representative of 6 samples/group. CPT-1a, DGAT2, CD36 and FATP2 protein expressions were normalized by α-tubulin. Data are means ± SEM. * *p* < 0.05, and ** *p* < 0.01 vs. the control cells. # *p* < 0.05 vs. PA group. CPT-1a: carnitine palmitoyl-transferase 1a; DGAT2: diacylglycerol *O*-acyltransferase 2; CD36: fatty acid-transporter, and FATP2: very long-chain acyl-CoA synthetase 1.

Regarding the fatty oxidation pathway, PA significantly increased CPT-1a protein expression. This effect did not occur in the case of cells treated with resveratrol and its metabolites (Figure 7a). To study the uptake of fatty acids across the plasma membrane, CD36 and FATP2 proteins were analyzed. After 18 h of treatment, no significant changes in CD36 were observed when hepatocytes treated with resveratrol or its metabolites were compared to PA cultures (Figure 7c). In the case of FATP2, R-3G, R-S and DH-R, these were able to reduce its protein expression (Figure 7d). Finally, to appraise the influence on triglyceride assembly, protein expression of DGAT2 was assessed, but no significant differences were observed (Figure 7b).

## 3. Discussion

Given that resveratrol shows significant positive effects on liver steatosis in spite of its low bioavailability, the aim of this study was to analyze whether phase II metabolites (R-4G, R-3G, R-S) [41,42,43] were effective in reducing hepatic fat accumulation. In addition, we also tested the potential activity of DH-R, the main microbial resveratrol metabolite [44,45]. The reason why we approached the subject of the resveratrol metabolites activity with an in vitro experimentation model is that the commercially available price and amount of these compounds are incompatible with an in vivo experimentation.

We found that resveratrol, and all the analyzed metabolites, at 1 µM partially prevented lipid accumulation induced by the saturated fatty acid PA in AML12 hepatocytes. When higher concentrations were used, only 10 µM DH-R and 25 µM R-4G induced significant effects. However, several authors have reported positive effects of resveratrol at higher concentrations (10–100 µM) in in vitro assays [46,47]. Indeed, Ji et al. (2015) reported a reduction in triglyceride levels in AML12 cells from 25 to 100 μM of resveratrol. However, this range of resveratrol concentrations is supraphysiological. Moreover, the experimental conditions performed by Ji et al. are not the same as those of the present study. Ji et al. used a methionine and choline-deficient medium to induce hepatic steatosis instead of palmitic acid, and they incubated the cells during 24 h, instead of 18 h [48]. In addition, Zhang et al. (2015) observed that treatment with resveratrol (10–80 µM) for 24 h prompted a dose-dependent reduction in triglycerides in steatotic HepG2 hepatocytes [49]. Moreover, Wang et al. (2012) also observed a decrease in lipid content by using primary mice hepatocytes incubated with oleic acid and 50 µM of resveratrol for 12 h [50]. Ardid-Ruiz et al. (2019) recently reported that incubation of steatotic HepG2 cells with resveratrol (1–10 µM) for 24 h significantly reversed the lipid accumulation to levels of untreated cells; however, this effect was not observed at the highest dose of resveratrol used (50 µM) [51]. The discrepancy between the reported results and those found in the present study may be due to differences in the experimental model. Indeed, most research is focused on the positive effects of resveratrol in the frequently used HepG2 experimental model. HepG2 is a hepatocellular carcinoma cell line with a gene expression profile that highly correlates with primary hepatocellular carcinoma tumors [52]. By contrast, nontumoral AML12 cells present an energy metabolism closer to primary hepatocytes [53].

The surprising fact that a lower dose was more effective than a higher one had been previously observed for resveratrol in in vivo experiments devoted to analyzing the effects of this polyphenol on obesity, carried out by our group [54] and other authors [55]. Thus, the study carried out by Cho et al. goes in the same line as our study. The authors reported that, in an in vivo model of steatosis in diet-induced obese mice, a lower dose of resveratrol was more effective than a higher one [55]. Moreover, in our study addressed in rats treated with resveratrol at doses of 6, 30, and 60 mg/kg/d, we also observed that the dose of 30 mg/kg/d was more effective in reducing adipose tissue size than 60 mg/kg/d [54]. The reason for this fact is not clear, and further studies are required. Nevertheless, it could be proposed that in the present study, R 1 μM is the most effective dose because it could be metabolized in R-3G (which seems to be the major metabolite formed) to a higher extent than higher concentrations such as R 10 or 25 μM. At these higher doses, the glucuroniltransferase (UGT) of AML12 cells could be saturated. In fact, the glucuronidation pathway seems to be a saturable metabolic pathway [43]; specifically, it has been described that the UGT1A7 and UGT1A10 enzymes are saturable pathways, while the sulfation is not [56]. Finally, it is very important to emphasize the fact that both resveratrol and its metabolites were active at 1 μM, since maximum blood concentrations of this phenolic compound after oral consumption tend to be around 1 µM [6,57,58].

In view of the effectiveness of the main resveratrol metabolites as anti-steatotic molecules in hepatocytes, it can be proposed that the effect on liver steatosis observed in in vivo studies using animal models, is not exclusively due to the parent compound, but also to its metabolites. Indeed, both phase II metabolites and DH-R, which is the main metabolite produced by gut microbiota, can contribute to the effects of its parent compound. The extent to which each metabolite exerts influence in the anti-steatotic effect of resveratrol will depend on the amount produced. Thus, as far as phase II metabolites are concerned, resveratrol is almost exclusively glucuronated at position 3 in rat and mice liver microsomes. In a similar way, in other species such as humans and dogs resveratrol is also glucuronidated at position 4′ (ratio R-3G:R-4G = 5:1) [59]. Moreover, sulphate metabolites are more abundant in humans than in rodents [9,51,60]. This may explain why resveratrol administration is effective despite of its low bioavailability. Therefore, it can be stated that the limitation in the use of this phenolic compound as an active biomolecule for fatty liver management due to its low bioavailability is not as important as it was thought.

When hepatocytes were incubated with resveratrol, it was not possible to assure that the reduction in triglyceride accumulation was due to this compound, because during the incubation period it could be metabolized to some extent. Consequently, in order to gain a deeper insight into this issue, additional experiments on resveratrol metabolism were carried out. Thus, the quantification of resveratrol in the incubation medium and inside the hepatocytes showed that it disappeared over time. In fact, after 6–8 h of incubation, no resveratrol was found in the medium, while the metabolite R-3G did appear. This data is in good accordance with the presence of R-3G quantified inside the cells from 1 h of incubation. Moreover, when β-glucuronidase was added to the incubation medium, R-3G proved to have a lesser presence. Taken together, these results confirm that resveratrol easily crosses the AML12 hepatocyte plasma membrane. It then suffers rapid phase II metabolic reactions inside the cells that conjugate the parent compound with glucuronide moieties. R-3G also crosses the hepatocyte plasma membrane towards the incubation medium. In line with these results, Delmas et al. (2013) reported that resveratrol could enter the cells via either passive diffusion or a facilitated process by lipid-rafts mediated endocytosis [61]. In addition, Ardid-Ruiz et al. (2019) showed the complete metabolism of resveratrol (10 µM) into to R-3S after 24 h of incubation with human HepG2 hepatocytes [51]. The variation in the quantified resveratrol metabolite is due to the well-documented differences in resveratrol metabolism between humans and rodents; indeed, glucuronidation in position 3 represents a major metabolic pathway in mice and rats [59,62].

Finally, hepatocytes were incubated with resveratrol alone or with β-glucuronidase, and the amount of triglycerides accumulated inside hepatocytes was measured. We observed that, in presence of β-glucuronidase, triglyceride accumulation induced by PA was not as well countered as it was in the absence of enzyme. These results suggest that R-3G can, in fact, be even more effective in preventing hepatocyte triglyceride accumulation than its parent compound, resveratrol. In some cases, the effect observed in cells treated with resveratrol are lower than those found in cells treated with R-3G. This can be due to the fact that, in cells treated with resveratrol, although at 18 h the parent compound was totally metabolized to R-3G, during the first 6–8 h both resveratrol and R-3G coexisted.

In the present study, we also analyzed the effects of resveratrol and its metabolites on the main metabolic pathways involved in triglyceride accumulation. Our results revealed that the incubation of hepatocytes with PA induced a clear increase in FAS activity. Moreover, CPT-1a protein expression was also significantly increased in steatotic AML12 hepatocytes. Consequently, it can be proposed that, in hepatocytes incubated with PA for 18 h, the up-regulation of β-oxidation is a compensatory mechanism aimed at increasing the transport of fatty acids into the mitochondrion for their subsequent oxidation, while minimizing steatosis development. This phenomenon has also been observed in an in vivo study carried out by our research group, by using a model of liver steatosis induced by high-fat high-fructose feeding (data submitted). With regard to the phenolic compounds, resveratrol, as well as all its metabolites, avoided the increase in FAS activity, the metabolites being even more efficient than the parent compound. Consequently, in hepatocytes treated with these molecules, CPT-1a was not increased, on the grounds that a compensatory mechanism was no longer necessary. Additionally, in the case of ACC protein, glucuronide metabolites also inhibited this enzyme. As a whole, our results show that the partial prevention of steatosis induced by resveratrol and its metabolites was due, at least in part, to their effects on *de novo* lipogenesis.

Regarding the transport of fatty acid into the hepatocytes, it has been described that this lipid species enters cells by both passive diffusion (flip-flop) and by using hepatic fatty acid transporters, such as CD36 and FATP2 [63]. In the present study, we observed that three resveratrol metabolites, co-incubated with the fatty acid PA, reduced fatty acid uptake because they decreased FATP2 protein expression. These results show that in the case of these metabolites, reduced fatty acid availability for triglyceride synthesis was not only due to the inhibition of *de novo* lipogenesis, but also to the reduction in the uptake from blood stream. Finally, it could be suggested that no changes in the triglyceride assembly were induced by any of the treatments used in the present study, since DGAT2 protein expression was not modified.

## 4. Conclusions

In summary, this is the first time that the anti-steatotic effect of phase II resveratrol metabolites and the microbial resveratrol metabolite, dihydroresveratrol, are evidenced. This is very significant information because it demonstrates that resveratrol metabolites contribute to the beneficial effect of the parent compound on fatty liver. It further suggests that the low bioavailability of resveratrol is not such an issue, as it was previously thought. The anti-steatotic effect was mainly due to the inhibition of *de novo* lipogenesis.

## 5. Materials and Methods

### 5.1. Reagents

Dulbecco modified Eagles minimal essential medium (DMEM)/HAM’s F12 Glutamax, and insulin, transferrin, and selenium (ITS) were obtained from Thermofisher (Waltham, MA, USA). Foetal bovine serum (FBS) was purchased from Corning (New York, NY, USA). Streptomycin-penicillin solution and trypsin/EDTA were obtained from Lonza (Basel, Switzerland). β-glucuronidase type HP-2, acetyl-coenzyme A, dexamethasone, malonyl coenzyme A, NADPH, palmitic acid (PA), resveratrol (≥99%), methanol, acetonitrile, and formic acid (FA) were all purchased from Sigma-Aldrich (St Louis, MO, USA). *Trans*-resveratrol-4′-*O*-D-glucuronide (≥98%), *trans*-resveratrol-3-*O*-β-D-glucuronide (≥95%), *trans*-resveratrol-3-*O*-sulfate (≥95%), dihydroresveratrol (≥98%) were all obtained from Cayman Chemical (Ann Arbor, MI, USA).

### 5.2. Cell Culture and Maintenance

Mouse hepatocyte AML12 (alpha mouse liver 12; ATCC^®^ CRL-2254™) were obtained from ATCC (Manassas, VA, USA). These cells were maintained in 75 cm2 flasks in DMEM/HAM’s F12 Glutamax supplemented with 10% heat inactivated fetal bovine serum plus 5 µg/mL insulin, 5 µg/mL transferrin, 5 ng/mL selenium, 40 ng/mL dexamethasone, and 1% penicillin/streptomycin (10,000 U/mL). AML12 were grown at 37 °C in humidified atmosphere with 5% CO_2_. When the cell monolayer reached 75% of confluence, cells were detached with a solution of trypsin-EDTA, and then harvested to perform subsequent experiments.

### 5.3. Experimental Design

An in vitro model mimicking the hepatocyte situation in fatty liver was created by using mouse AML12 cells, which were grown in 6-well plates and incubated with 0.3 mM of palmitic acid (PA) for 18 h to induce triglyceride accumulation. To achieve the appropriate conditions for this in vitro study (toxicity and triglyceride levels) a time-course (6, 18, 24, and 48 h) by using 0 to 500 μM of PA was performed. The results showed that at 18 h and at 300 μM of PA, the hepatocytes accumulated the highest amount of triglyceride, without compromising cell viability to a high extent. Then hepatocytes were co-incubated with or without PA (300 μM) and resveratrol or its metabolites (R-4G, R-3G, R-S and DH-R) at 1, 10, or 25 µM (diluted in 95% ethanol), because these doses are in the range of those used in the vast majority of the reported studies devoted to analyzing the potential beneficial effects of resveratrol and its metabolites. It is important to emphasize that the lowest dose (1 μM) is similar to the maximum blood concentrations of these metabolites after oral administration of resveratrol at different doses [6,57,58]. In the case of the control group, the same volume of that of the vehicle was used. After 18 h, cells were used for the subsequent experiments. Each experiment was performed at least three times.

### 5.4. Triacylglycerol Levels Determination

After treatment, the medium was removed and cell extracts were used for triglyceride determination. AML12 cells were washed extensively with phosphate-buffered saline (PBS), and the suspension was sonicated in 10 mM Tris-HCl pH 7.4, 150 mM NaCl and 1 mM EDTA on ice with a 5-s burst in a Branson Sonifier SFX550 (Saint Louis, MO, USA) fitted with a microtip. Subsequently, triglyceride content was measured with a commercial kit (Spinreact, Girona, Spain). Protein measurements were performed using the Bradford method [64]. Triglyceride content values were obtained as mg triglycerides/mg protein, and expressed as the percentage of the control cells.

### 5.5. Cell Viability Assay

The live cell number was evaluated with the crystal violet assay based on the cell staining with crystal violet [65]. Briefly, AML12 cells were seeded to 96-well tissue culture plates at 5 × 10^3^ cells per well. Three days after plating the cells, these were treated with the pertinent compounds for 18 h. After treatments, cells were washed with PBS, fixed in 3.7% formaldehyde, and stained with 0.25% crystal violet for 20 min in the dark. Finally, the resulting crystals were solubilized with 33% acetic acid, and the absorbance was registered at 590 nm in an iMark microplate reader (Bio-Rad, Hercules, CA, USA). Cell viability was expressed as the percentage of the control cells.

### 5.6. Optical Microscopy Analysis of Ateatotic AML12 Hepatocytes

Lipid droplet accumulation was analyzed by optical microscopy. AML12 cells were seeded in 6-well culture plates and incubated with their respective treatments for 18 h photographed under an Olympus CH optical microscope (Olympus, Tokyo, Japan) and examined with a 40× objective. The cell features were analyzed by ImageJ software (NIH, Bethesda, MD, USA).

### 5.7. UHPLC Analysis of AML12 and Cell Media

Hepatocytes media supernatants were collected, centrifuged for 20 min (20,000 *g*, 4 °C) before being analyzed by ultra-high-performance liquid chromatography (UHPLC) to determine cellular metabolism of resveratrol in cell culture. Moreover, quantification of phenolic compounds was also carried out inside the cells. For this purpose, AML12 cells were washed with ice-cold PBS, and lysed with methanol. After evaporation of cellular extracts with liquid N_2_, the samples were dissolved in methanol/H_2_O (50/50, *v*/*v*) and centrifuged for 20 min (20,000 *g*, 4 °C) before UHPLC analysis.

Samples were analyzed using a 1290 Infinity UPLC (Agilent Technologies, Courtaboeuf, France). Two µL were injected into an Agilent SB-C18 column (1.8 µm, 2.1 × 100 mm). Samples were eluted with solvent A (H_2_O 0.1% FA) and solvent B (acetonitrile 0.1% FA) by the following gradient program: 0–1.7 min, 10% B; 1.7–3.4 min, 10–20% B; 3.4–5.1 min, 20–30% B; 5.1–7.8 min, 30% B; 7.8–8.5 min, 30–35% B; 8.5–11.9 min, 35–60% B; 11.9–15.3 min, 60–100% B; 15.3–17 min, 100% B; 17–17.3 min, 100–10% B. The flow rate was set to 0.4 mL/min and the UV detector was set at the wavelength 306 nm.

### 5.8. Protein Immunodetection

Phospho-acetyl-CoA carboxylase (p-ACC), total acetyl-CoA carboxylase (total ACC), fatty acid synthase (FAS), CD36 molecule (CD36), carnitine palmitoyltransferase 1-a (CPT1-a), solute carrier family 27 member 2 (FATP2), diacylglycerol O-acyltransferase 2 (DGAT2), and α-tubulin were detected by western blot. Cellular protein extracts were denaturalized at 95 °C for 5 min in Laemmli buffer [66] and separated by SDS-PAGE electrophoresis in 4–15% polyacrylamide gels. Gels were transferred onto PVDF membranes by electroblotting with constant amperage (1 mA/cm^2^). After blocking for 1 h at room temperature, membranes were incubated overnight at 4 °C with the corresponding primary antibody (anti-p-ACC 1:1000, anti-total ACC 1:1000, anti-FAS 1:1000, anti-CD36 1:1000, anti-CPT1-a 1:1000, anti-FATP2 1:1000, anti-DGAT2 1:1000, and anti- α-tubulin 1:2000). After washing, membranes were probed with the secondary antibody conjugated to horseradish peroxidase. The immunoreactive proteins were detected by the Forte Western HRP substrate (Millipore; Burlington, MA, USA), and the blots were imaged by scanning with the ChemiDoc™ MP Imaging System (Bio-Rad, Hercules, CA, USA). α-Tubulin was used as the loading control.

### 5.9. Fatty Acid Synthase (FAS) Activity

The samples for assaying the lipogenic fatty acid synthase activity were centrifuged at 5000 *g* for 5 min at 4 °C. Thus, the supernatant fraction was used for quantification of enzyme activity and fatty acid synthase from the rate of malonyl-CoA dependent NADPH oxidation [67]. NADPH was measured by reading absorbance at 340 nm in an iEMS microplate reader (Lab systems; Bradenton, FL, USA). The enzyme assay was conducted at 37 °C. Soluble protein in the supernatant fraction was determined using bovine serum albumin as standard [64]. FAS activity was expressed as nmol NADPH consumed/min per mg protein.

### 5.10. Statistical Analysis

Data were expressed as mean ± standard error of the mean (SEM) from at least three independent experiments. The normality of the data was tested using the Shapiro–Wilk test. Comparisons between the control cells and the cells treated with each molecule, as well as between the cells treated with PA alone and PA together with resveratrol or its metabolites, were conducted by Student’s t test or Mann–Whitney’s U test as appropriate, using the statistical package SPSS 19.0 (SPSS Inc., Chicago, IL, USA). Differences between means were considered significant at *p* < 0.05.

## Abbreviations

AML12	alpha mouse liver 12 cell line
CD36	CD36 molecule
CPT1-a	carnitine palmitoyltransferase 1-a
DMEM/HAM’s F12	Dulbecco modified Eagles minimal essential medium/HAM’s F12 Glutamax
DGAT2	diacylglycerol *O*-acyltransferase 2
DH-R	dihydro-resveratrol
EDTA	ethylenediamine tetra acetate
FAS	fatty acid synthase
FATP2	solute carrier family 27 member 2
HRP	horseradish-peroxidase
NADPH	reduced nicotinamide adenine dinucleotide phosphate
PA	palmitic acid
p-ACC	phospho-acetyl-CoA carboxylase
PBS	phosphate-buffered saline
PVDF	polyvinylidene difluoride
R-4G	*trans*-resveratrol-4′-*O*-glucuronide
R-3G	*trans*-resveratrol-3-*O*-glucuronide
R-S	*trans*-resveratrol-3-*O*-sulfate
SDS-PAGE	sodium dodecyl sulphate polyacrylamide gel electrophoresis
total ACC	total acetyl-CoA carboxylase
UHPLC	ultra-high-performance liquid chromatography
